# Radiation Facility Volume and Survival for Men With Very High-Risk Prostate Cancer Treated with Radiation and Androgen Deprivation Therapy

**DOI:** 10.1001/jamanetworkopen.2023.27637

**Published:** 2023-08-08

**Authors:** Nikhil Sebastian, Subir Goyal, Yuan Liu, James R. Janopaul-Naylor, Pretesh R. Patel, Vishal R. Dhere, Sheela Hanasoge, Jay W. Shelton, Karen D. Godette, Ashesh B. Jani, Bruce Hershatter, Benjamin Fischer-Valuck, Sagar A. Patel

**Affiliations:** 1 Department of Radiation Oncology, Winship Cancer Institute, Emory University, Atlanta, Georgia; 2 Biostatistics and Bioinformatics Shared Resource, Winship Cancer Institute, Emory University, Atlanta, Georgia; 3 Department of Biostatistics and Bioinformatics, Emory University, Atlanta, Georgia; 4 Radiation Oncology at Swedish, Englewood, Colorado; 5 Department of Urology, Emory University, Atlanta, Georgia

## Abstract

**Importance:**

Very high-risk (VHR) prostate cancer is an aggressive substratum of high-risk prostate cancer, characterized by high prostate-specific antigen levels, high Gleason score, and/or advanced T category. Contemporary management paradigms involve advanced molecular imaging and multimodal treatment with intensified prostate-directed or systemic treatment—resources more readily available at high-volume centers.

**Objective:**

To examine radiation facility case volume and overall survival (OS) in men with VHR prostate cancer.

**Design, Setting, and Participants:**

A retrospective cohort study was performed from November 11, 2022, to March 4, 2023, analyzing data from US facilities reporting to the National Cancer Database. Patients included men diagnosed with nonmetastatic VHR prostate cancer by National Comprehensive Cancer Network criteria (clinical T3b-T4 category, primary Gleason pattern 5, >4 cores with grade group 4-5, and/or 2-3 high-risk features) and treated with curative-intent radiotherapy and androgen deprivation therapy between January 1, 2004, to December 31, 2016.

**Exposures:**

Treatment at high- vs low-average cumulative facility volume (ACFV), defined as the total number of prostate radiotherapy cases at an individual patient’s treatment facility from 2004 until the year of their diagnosis. The nonlinear association between a continuous ACFV and OS was examined through a Martingale residual plot; an optimal ACFV cutoff was identified that maximized the separation between high vs low ACFV via a bias-adjusted log rank test.

**Main Outcomes and Measures:**

Overall survival was assessed between high vs low ACFV using Kaplan-Meier analysis with and without inverse probability score weighted adjustment and multivariable Cox proportional hazards.

**Results:**

A total of 25 219 men (median age, 71 [IQR, 64-76] years; 78.7% White) with VHR prostate cancer were identified, 6438 (25.5%) of whom were treated at high ACFV facilities. Median follow-up was 57.4 (95% CI, 56.7-58.1) months. Median OS for patients treated at high ACFV centers was 123.4 (95% CI, 116.6-127.4) months vs 109.0 (95% CI, 106.5-111.2) months at low ACFV centers (*P* < .001). On multivariable analysis, treatment at a high ACFV center was associated with lower risk of death (hazard ratio, 0.89; 95% CI, 0.84-0.95; *P* < .001). These results were also significant after inverse probability score weighted–based adjustment.

**Conclusions and Relevance:**

In this cohort study of patients with VHR prostate cancer who underwent definitive radiotherapy and androgen deprivation therapy, facility case volume was independently associated with longer OS. Further studies are needed to identify which factors unique to high-volume centers may be responsible for this benefit.

## Key Points

**Question:**

Is there a difference in outcomes between patients with very high-risk prostate cancer who are treated at radiation facilities with high vs low patient volume?

**Findings:**

In this cohort study of 25 219 men in the US with very high-risk prostate cancer treated with radiotherapy and hormone therapy, treatment at a radiation facility with high annual volume of similar cases was independently associated with improved overall survival. This association was significant for academic and nonacademic centers alike.

**Meaning:**

These findings suggest that the expertise and resources that accompany high-volume treatment facilities are associated with improved outcomes for men with very high-risk prostate cancer, but further investigation is needed to identify the specific causes for this association.

## Teaser

This cohort study examines the overall survival in men with very high-risk prostate cancer who are treated with radiotherapy and hormone therapy at radiation facilities with a higher vs lower volume of cases per year.

## Introduction

Prostate cancer remains the most common cancer in men, accounting for more than 20% of incident cancer cases in US men in 2022.^1^ In 2012, the US Preventive Services Task Force recommended against routine prostate-specific antigen (PSA) screening owing to risk of overdiagnosis of clinically insignificant prostate cancer. Consequently, there has been a decrease in indolent, localized disease, but a concordant increase of advanced prostate cancer diagnoses^2,3,4^ as well as an increase in prostate cancer mortality.^5^ Very high-risk (VHR) prostate cancer is a substratum comprising men with nonmetastatic clinical category T3b or T4, primary Gleason pattern 5 on biopsy, more than 1 high-risk feature, or more than 4 cores of Gleason 8 to 10 disease. The risk of distant metastasis after treatment for men with VHR prostate cancer is particularly higher than that of men with high-risk disease as a whole.^6,7,8^

While radiation plus androgen deprivation therapy (ADT) remains a first-line treatment option for VHR prostate cancer, the optimal paradigm is rapidly evolving.^9^ For example, radiation can be delivered with external beam radiotherapy (EBRT) alone or EBRT with a brachytherapy boost. A randomized clinical trial^10^ and multi-institutional retrospective data^11,12^ have reported improved cancer outcomes with EBRT plus a brachytherapy boost compared with EBRT alone. Furthermore, for men undergoing radiotherapy plus ADT, randomized trials have shown improved survival outcomes with systemic treatment intensification with addition of a novel androgen-signaling inhibitor, such as abiraterone, or with docetaxel added to standard ADT.^7,8,13^ As such, treatment for these patients with VHR prostate cancer may be nuanced, complex, and resource intensive—features that may be more readily navigated at high-volume cancer centers.

Numerous studies of patients with other aggressive cancer types have observed that treatment at high-volume facilities is associated with higher rates of long-term overall survival (OS), including those who undergo primary surgery, radiotherapy, or chemotherapy.^14,15,16,17,18,19,20,21,22,23,24^ Whether radiotherapy case volume influences long-term outcomes in men with VHR prostate cancer is unknown. Herein, we examine whether radiation facility case volume is associated with 10-year survival of men with VHR prostate cancer treated with definitive radiotherapy within Commission on Cancer–accredited facilities in the US. Given the complexity of management of this group of men with prostate cancer, we hypothesized that men treated at high-volume centers will have improved OS compared with those treated at low-volume centers.

## Methods

### Data Source and Study Population

The National Cancer Data Base (NCDB), a nationwide hospital-based cancer registry jointly sponsored by the American College of Surgeons and the American Cancer Society, collects data from more than 1500 Commission on Cancer–accredited hospitals and captures approximately 70% of incident cancer cases in the US annually.^25^ Data accuracy and quality are continually validated via data quality review, site surveys, and internal monitoring.^26^ Because this study used deidentified data from the NCDB, the requirement for formal institutional review and the need for informed consent were waived, consistent with the policies of Emory University School of Medicine, Atlanta, Georgia. The study followed the Strengthening the Reporting of Observational Studies in Epidemiology (STROBE) reporting guideline for cohort studies.

Using the NCDB, we identified men diagnosed with VHR prostate adenocarcinoma treated with curative-intent radiotherapy (ie, external-beam radiation with or without brachytherapy boost) and concomitant ADT between January 1, 2004, and December 31, 2016. Very high-risk prostate cancer was defined by National Comprehensive Cancer Network criteria,^9^ which included patients with clinical category T3b or T4, primary Gleason pattern 5 on biopsy, more than 1 NCCN high-risk feature, or more than 4 cores of Gleason 8 to 10 disease. To encompass both moderately hypofractionated and standard fractionated schedules and exclude incomplete or palliative courses of radiotherapy, only those who received a total radiation dose of 60 Gy or more for external beam radiation alone or 37.5 Gy or more for external beam radiation plus brachytherapy boost were included in the analysis. Patients with unknown tumor stage, biopsy Gleason score, or PSA, or who underwent upfront radical prostatectomy were excluded. Men who initiated ADT more than 1 year before or after the start of radiotherapy were excluded. Patients whose radiotherapy was delivered at multiple facilities or whose facility information was unknown were also excluded.

### Defining High vs Low Treatment-Volume Facilities

All facilities delivering prostate radiotherapy, before we applied exclusion and inclusion criteria, were included in our initial analysis and the number of prostate radiotherapy cases for each facility per year was calculated. Given that a facility’s radiotherapy patient volume can vary from year to year, a cumulative facility volume was defined as the total number of radiotherapy cases at an individual patient’s treatment facility from 2004 until the year of diagnosis of the patient. This cumulative facility volume, specific to each patient, was then divided by the total number of years that the facility reported to the NCDB until that patient’s year of diagnosis and was subsequently defined as the average cumulative facility radiation volume (ACFV) for that individual patient. The ACFV is therefore defined at the level of the patient and represents the experience level of the treating facility at the time that specific patient was treated. It is therefore possible in this analysis for patients treated at the same facility to be included as treated at either a high ACFV center or low ACFV center based on the year of diagnosis of that individual patient and the case volume per year at that facility leading up to that patient’s treatment. The advantage of ACFV used in this analysis, as opposed to calculating a facility’s patient volume in aggregate across many years, is that it accounts for changes in facility volume and/or expertise that may occur over time during the 12-year study period. In the final analysis data, the nonlinear association between a continuous ACFV and OS was visualized in a Martingale residual plot, in which the Martingale residuals were estimated from the Cox proportional hazards model and then plotted against the ACFV by local a linear regression curve. The optimal ACFV cutoff point was identified that maximizes the separation between the 2 groups (high vs low ACFV) via a bias-adjusted log rank test.^27^ The method enables the estimation and evaluation of the significance of the cutoff value while controlling for the bias created by the data-driven searching process.

### Statistical Analysis

Descriptive statistics were used to present baseline characteristics. Covariates included facility type (academic vs nonacademic), age at diagnosis, race, insurance provider, residential median income, educational level (measured as the percentage of adults in the patient’s zip code without a high school degree), Charlson-Deyo Comorbidity Index score, T category, biopsy Gleason score, PSA level, radiation dose, use of chemotherapy, use of brachytherapy boost, year of diagnosis, and patient distance from treatment facility. Race was included as it has been independently associated with survival outcomes in men with prostate cancer and may confound the association between facility volume and OS. Cutoffs for stratification were selected for each covariate. For age and total radiation dose, the median was used for dichotomization. For distance to the treatment facility, quartiles were used. For income and high school degree, subgroups were prespecified in the NCDB. For year of diagnosis, cutoffs were determined based on major US policies that impacted prostate cancer management and/or NCDB data changes, such as inclusion of active surveillance data in 2010, United States Preventive Services Task Force grade D recommendation against PSA screening in 2012, and enactment of major provisions of the Affordable Care Act in 2014. Analysis of variance and the χ^2^ test were used to compare clinical and demographic characteristics between high- and low-volume facilities. The primary end point was OS, defined as months from the date of diagnosis to the date of death, with patients lost to follow-up censored at the date of last documented visit. Kaplan-Meier analysis with and without propensity score–based adjustment using inverse probability score weighting (IPSW) was used to compare OS between those treated at high- vs low-volume facilities. All measured covariates were used to estimate the propensity score. In the propensity score–weighted cohort, the balance of covariates between groups was evaluated by the standardized differences, and a value of less than 0.1 was considered a negligible imbalance. Multivariable Cox proportional hazards, built by backward variable selection procedure with an α of .05 removal criteria, were used to estimate hazard ratios (HRs) and 95% CIs between those who were treated at high- vs low-volume facilities. Analyses used SAS, version 9.4 (SAS Institute Inc) from November 11, 2022, to March 4, 2023. Tests were 2 sided, with a 0.05 level of significance.

## Results

**Figure 1.**
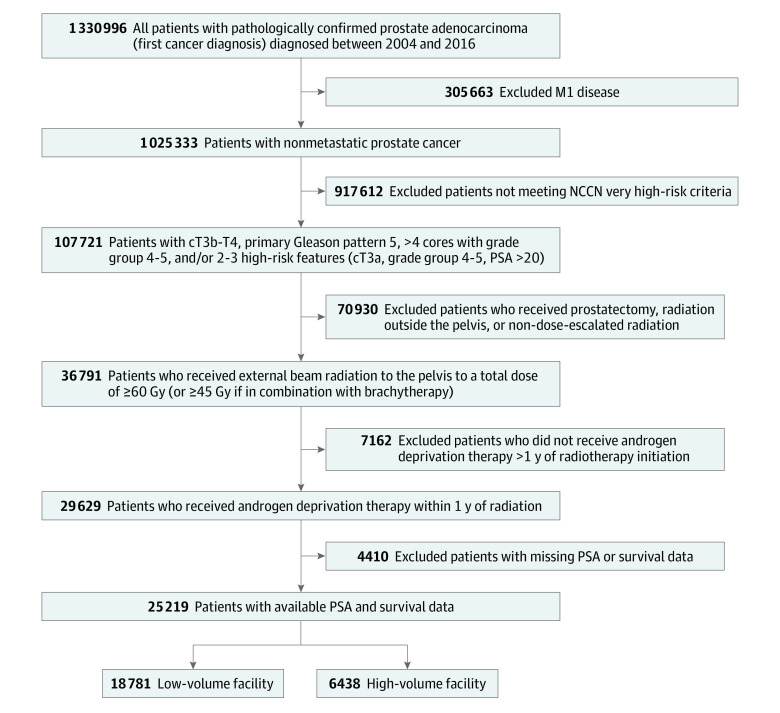
Diagram of Study Cohort M1 indicates bone metastases; NCCN, National Comprehensive Cancer Network; PSA, prostate-specific antigen.

Figure 1 shows the schema used to identify the eligible study cohort of 25 219 patients. Median follow-up was 57.4 (95% CI, 56.7-58.1) months. The median ACFV was 56 (IQR, 33-90) patients treated per year, and the optimal ACFV cutoff was 89 patients treated per year. A total of 6438 patients (25.5%) were treated at high ACFV centers, and 18 781 patients (74.5%) were treated at low ACFV centers. Compared with low ACFV centers, high ACFV centers were more often academic/research programs (49.2% vs 24.1%; *P* < .001) and more commonly treated patients with private insurance (32.3% vs 24.9%; *P* < .001). Overall median age was 71 (IQR, 54-76) years, and 78.7% of the patients were of White race. Patients treated at high-ACFV centers were younger (age ≥70 years, 48.9% vs 50.5%; *P* = .02), less frequently White race (77.2% vs 79.3%; *P* < .001), had fewer comorbidities (Charlson-Deyo Comorbidity Index score 0, 85.9% vs 81.9%; *P* < .001), and lived in areas with higher proportion of high school graduates (zip codes with <7% population without high-school degree, 28.1% vs 22.7%; *P* < .001) and higher median incomes (income≥$68 000 per year, 40.1% vs 28.2%; *P* < .001). Patient sociodemographic and clinical characteristics stratified by ACFV are summarized in Table 1. After IPSW adjustment, all baseline characteristics were distributed evenly with an absolute standard difference less than 0.1 between high and low ACFV centers (eTable 1 in Supplement 1).

**Table 1.** Patient Characteristics for High vs Low Average Cumulative Facility Volume

Covariate and level information	Patients, No. (%)			*P* value^a^
	Total (N = 25 219)	Average cumulative facility volume		
		Low (n = 18 781 [74.5%])	High (n = 6438 [25.5%])	
Facility type				
Nonacademic/research program	17 522 (69.5)	14 249 (75.9)	3273 (50.8)	<.001
Academic/research program	7697 (30.5)	4532 (24.1)	3165 (49.2)	
Age, y				
<70	12 578 (49.9)	9289 (49.5)	3289 (51.1)	.02
≥70	12 641 (50.1)	9492 (50.5)	3149 (48.9)	
Race				
Black	4311 (17.1)	3198 (17.0)	1113 (17.3)	<.001
White	19 859 (78.7)	14 892 (79.3)	4967 (77.2)	
Other^b^	1049 (4.2)	691 (3.7)	358 (5.6)	
Zip code median income, $				
≥68 000	7876 (31.2)	5292 (28.2)	2584 (40.1)	<.001
48 000-67 999	6641 (26.3)	5137 (27.4)	1504 (23.4)	
38 000-47 999	5916 (23.5)	4648 (24.7)	1268 (19.7)	
<38 000	4693 (18.6)	3634 (19.3)	1059 (16.4)	
Zip code without high school degree, %				
<7.0	6046 (24.0)	4245 (22.7)	1801 (28.1)	<.001
7.0-12.9	8361 (33.2)	6208 (33.1)	2153 (33.4)	
13.0-20.9	6569 (26.0)	5026 (26.8)	1543 (24.0)	
≥21	4171 (16.5)	3250 (17.43	921 (14.3)	
Insurance type				
Medicaid/uninsured	2545 (10.1)	2060 (11.0)	485 (7.5)	<.001
Private	6756 (26.8)	4679 (24.9)	2077 (32.3)	
Medicare	15 918 (63.1)	12 042 (64.1)	3876 (60.2)	
Charlson-Deyo Comorbidity Index score				
0	20 907 (82.9)	15 375 (81.9)	5532 (85.9)	<.001
1	3322 (13.2)	2628 (14.0)	694 (10.8)	
≥2	990 (3.9)	778 (4.1)	212 (3.3)	
AJCC Clinical T category				
T1	9351 (37.1)	7132 (38.0)	2219 (34.5)	<.001
T2	9052 (35.9)	6800 (36.2)	2252 (35.0)	
T3-4	6816 (27.0)	4849 (25.8)	1967 (30.6)	
PSA, ng/mL				
<10	10 225 (40.5)	7534 (40.1)	2691 (41.8)	.03
10-20	5413 (21.5)	4027 (21.4)	1386 (21.5)	
>20	9581 (38.0)	7220 (38.4)	2361 (36.7)	
Gleason score				
6-7	1571 (6.2)	1085 (5.8)	486 (7.5)	<.001
8-10	23 483 (93.1)	17 571 (94.2)	5912 (91.8	
Total radiation dose, Gy				
<74	4995 (19.8)	3352 (17.8)	1643 (25.5)	<.001
≥74	20 224 (80.2)	15 429 (82.2)	4795 (74.5)	
Chemotherapy				
No	25 011 (99.2)	18 643 (99.3)	6368 (98.9)	.007
Yes	208 (0.8)	138 (0.7)	70 (1.1)	
Brachytherapy boost				
No	22 707 (90.0)	17 379 (92.5)	5328 (82.8)	<.001
Yes	2512 (10.0)	1402 (7.5)	1110 (17.2)	
Radiation treatment volume				
Unknown	2741 (10.9)	2261 (12)	480 (7.5)	<.001
Prostate with whole pelvis	11 675 (46.3)	8508 (45.3)	3167 (49.2)	
Prostate only	10 803 (42.8)	8012 (42.7)	2791 (43.4)	
Radiation modality				
3DCRT	931 (3.7)	668 (3.6)	263 (4.1)	<.001
IMRT	16 915 (67.1)	12 829 (68.3)	4086 (63.5)	
Proton	160 (0.6)	51 (0.3)	109 (1.7)	
Unknown	7213 (28.6)	5233 (27.9)	1980 (30.8)	
Year of diagnosis				
≥2004 to ≤2009	12 125 (48.1)	9211 (49.0)	2914 (45.3)	<.001
>2009 to ≤2011	10 353 (41.1)	7309 (38.9)	3044 (47.3)	
>2011 to ≤2014	8758 (34.7)	6688 (35.6)	2070 (32.2)	
>2014 to ≤2016	3555 (14.1)	2892 (15.4)	663 (10.3)	
Distance to facility (miles)				
≥0 to ≤4.1	6302 (25.1)	4983 (26.5)	1319 (20.5)	<.001
>4.1 to ≤9.1	6372 (25.3)	4704 (25.0)	1668 (25.9)	
>9.1 to ≤19.8	6197 (24.6)	4545 (24.2)	1652 (25.7)	
>19.8 to ≤2914.7	6285 (24.9)	4504 (24.0)	1781 (27.7)	

Abbreviations: AJCC, American Joint Committee on Cancer; 3DCRT, 3-dimensional conformal radiotherapy; IMRT, intensity-modulated radiation therapy; PSA, prostate-specific antigen.

SI conversion: To convert PSA to μg/L, multiply by 1.0.

^a^ The parametric *P* value was calculated by the χ^2^ test.

^b^ Other includes Native American, Asian, Pacific Islander, or unknown race; groups were collapsed because of small sample sizes.

**Figure 2.**
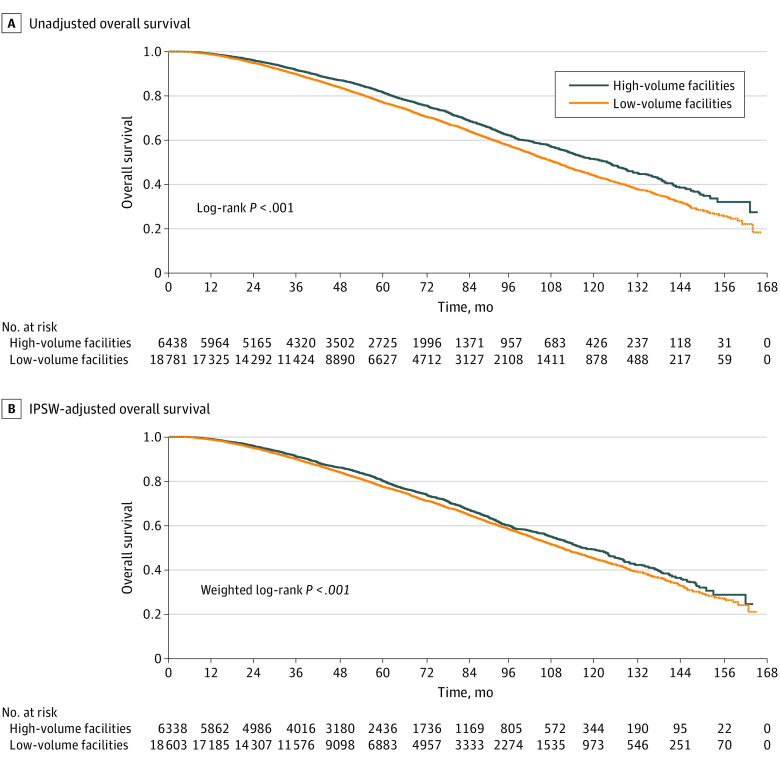
Overall Survival of Patients Treated at High vs Low Average Cumulative Facility Radiation Volume Centers Unadjusted (A) and inverse probability score weighting (IPSW)-adjusted (B) overall survival.

The median OS for patients treated at high-ACFV centers was 123.4 (95% CI, 116.6-127.4) months vs low-ACFV centers, which was 109.0 (95% CI, 106.5-111.2) months (log-rank *P* < .001) (Figure 2A). The estimated 10-year OS for patients treated at high-ACFV centers was 51.5% (95% CI, 49.1%-53.8%) vs low-ACFV centers, which was 43.9% (95% CI, 42.4%-45.4%). This OS benefit was significant after IPSW adjustment (Figure 2B).

On multivariable analysis, treatment at a high ACFV center was associated with a lower risk of death (HR, 0.89; 95% CI, 0.84-0.95; *P* < .001) compared with treatment at a low ACFV center. Other covariates on multivariable modeling that were associated with improved OS included younger age, race (White vs Black or other), higher income, private insurance, academic/research treatment facility, lower comorbidity, lower T category, lower PSA level, and lower Gleason score (Table 2). On IPSW-weighted analysis, treatment at high ACFV centers was associated with a lower risk of death (HR, 0.90; 95% CI, 0.85-0.95; *P* < .001). In the IPSW-adjusted multivariable analysis, treatment at a high-ACFV center was again associated with higher OS (HR, 0.90; 95% CI, 0.85-0.95; *P* < .001). The covariates that were associated with improved OS in the unadjusted model were also significant in the IPSW-adjusted model; additionally, a more recent year of diagnosis was associated with OS in the IPSW model (*P* = .04). With 2015-2016 as referent, HR was 1.00 (95% CI, 0.84-1.19) for 2004-2009, 0.96 (95% CI, 0.80-1.14) for 2010-2011, and 0.89 (95% CI, 0.75-1.06) for 2012-2014 (*P* = .04).

**Table 2.** Multivariable Cox Proportional Hazards Model for Overall Survival^a^

Covariate and level information	Overall survival		
	HR (95% CI)	HR *P* value	Overall *P* value
Average cumulative facility volume (optimal cutoff)			
Low	1.12 (1.05-1.19)	<.001	<.001
High	1 [Reference]		
Age, y			
≤70	0.65 (0.61-0.68)	<.001	<.001
>70	1 [Reference]		
Race			
Black	1.24 (1.05-1.45)	.009	<.001
White	1.37 (1.18-1.59)	<.001	
Other^b^	1 [Reference]		
Zip code median income, $			
≥68 000	0.82 (0.75-0.88)	<.001	<.001
48 000-67 999	0.94 (0.87-1.01)	.11	
38 000-47 999	0.99 (0.91-1.07)	.75	
<38 000	1 [Reference]		
Insurance type			
Medicaid/uninsured	1.05 (0.96-1.16)	.27	<.001
Private	0.79 (0.74-0.85)	<.001	
Medicare	1 [Reference]		
Facility type			
Nonacademic/research program	1.12 (1.05-1.18)	<.001	<.001
Academic/research program	1 [Reference]		
Charlson-Deyo Comorbidity Index score			
0	0.57 (0.50-0.64)	<.001	<.001
1	0.77 (0.67-0.88)	<.001	
≥2	1 [Reference]		
AJCC clinical T-stage			
T1	0.80 (0.75-0.86)	<.001	<.001
T2	0.87 (0.81-0.93)	<.001	
T3-4	1 [Reference]		
PSA, ng/mL			
<10	0.82 (0.77-0.87)	<.001	<.001
10-20	1.00 (0.94-1.07)	.96	
>20	1 [Reference]		
Gleason score			
6-7	0.63 (0.56-0.70)	<.001	<.001
8-10	1 [Reference]		

Abbreviations: AJCC, American Joint Committee on Cancer; HR, hazard ratio; PSA, prostate-specific antigen.

SI conversion: To convert PSA to μg/L, multiply by 1.0.

^a^ Backward selection with an α level of removal of .05 was used. The following variables were removed from the model: distance to facility, zip code percentage without high school degree, year of diagnosis, total radiation dose, and radiation modality.

^b^ Other includes Native American, Asian, Pacific Islander, or unknown race; groups were collapsed because of small sample sizes.

Given the association of facility volume and facility type (academic vs nonacademic), we created a multivariable Cox proportional hazards model for OS that included an interaction term between facility volume and type. There was no significant interaction of facility volume with OS for treatment at an academic vs nonacademic center (*P* = .39 for interaction) (eTable 2 in Supplement 1). On Kaplan-Meier analysis of OS of subgroups based on facility volume and type, high-volume status was associated with OS improvement, suggesting an independent association of facility volume with OS regardless of facility type (eFigure in Supplement 1).

## Discussion

Our analysis showed an association between radiation facility volume and long-term outcomes in men with VHR prostate cancer undergoing radiotherapy and concomitant ADT. Specifically, treatment at a high-volume facility was associated with a lower mortality rate compared with treatment at a low-volume facility. These findings are similar to earlier studies in other cancers that have shown an association between hospital and/or physician volume and improved outcomes.^14,15,16,17,18,19,20,21,22,23,24^

There is substantial clinical heterogeneity in the natural history and overall prognosis of men with high-risk prostate cancer. Very high risk is a substratum of prostate cancer with more aggressive clinicopathologic features; although randomized clinical trials and most retrospective studies have not separately considered this group of patients, their risk of distant metastasis after treatment is substantially higher than that of men with high-risk disease as a whole.^28,29,30^ Specifically, men with VHR prostate cancer may have a 10% to 15% higher risk of prostate cancer mortality vs other high-risk patients.^30^ Radiotherapy plus ADT remains a first-line treatment option for VHR prostate cancer, yet the optimal paradigm is rapidly evolving.^9^ For example, the role of prostate-directed dose-intensifying brachytherapy or elective pelvic lymph node irradiation has been poorly defined. However, prostate-directed treatment intensification with a brachytherapy boost has been shown to prevent distant metastases and improve prostate cancer–specific mortality in men with high-risk prostate cancer with adverse clinicopathologic characteristics.^9,10,11^ In the current analysis, patients treated at a high-volume facility were more likely to receive a brachytherapy boost compared with patients treated at a low-volume facility. Furthermore, among patients with prostate cancer who undergo a brachytherapy boost, previously reported studies have shown that treatment by a high-volume physician portends improved long-term outcomes.^31^ Regarding elective pelvic lymph node irradiation, its use for men with prostate cancer undergoing definitive radiotherapy was highly variable in the absence of defined clinical trial protocols during the period of this analysis. However, for VHR prostate cancer, randomized data have shown improved metastasis-free survival with the addition of pelvic nodal radiotherapy. Yet, pelvic nodal treatment has been reported to confer significantly higher rates of grade 2 or greater gastrointestinal or genitourinary toxic effects of up to 10%.^32,33,34^ Delivery of more aggressive, but toxic, treatment may be more readily available at high-volume centers. High-volume centers had a higher frequency of prostate plus whole pelvis radiotherapy compared with low-volume centers, but a large number of patients in both cohorts had unknown treatment volumes, limiting interpretation.

Similarly, men with VHR prostate cancer are likely to be considered for systemic treatment intensification, such as use of docetaxel or a novel androgen-signaling inhibitor, such as abiraterone, which has been shown to improve cancer-specific survival.^7,12^ The landmark trial (RTOG 0521) that showed a clinical benefit with the addition of docetaxel to radiotherapy plus ADT was active during the study period of this analysis, and initial results were presented in 2015.^8^ Indeed, patients treated at high-volume centers were significantly more likely to receive chemotherapy in our analysis, but few patients were prescribed chemotherapy overall. Furthermore, the NCDB does not report duration or drug of ADT, so whether patients received second-generation androgen-signaling inhibitors is unknown. Regardless, the survival advantage of high-volume facilities persisted even after adjustment for brachytherapy boost, radiotherapy fields, ADT, and chemotherapy use, so facility features independent of prescribed treatment appear to influence long-term outcomes for men with VHR prostate cancer.

Several other features of high-volume facilities, not directly quantifiable in the NCDB and unable to be assessed in this analysis, may explain our findings. First, radiation facility volume may correlate with increased expertise in treatment planning and delivery. Specifically, there was rapid evolution of radiotherapy technology during the study period, with transition from historic 3-dimensional conformal radiotherapy to intensity-modulated radiation therapy. Intensity-modulated radiation therapy incorporates tighter treatment margins with steep dose gradients, which help minimize toxicity but can risk undertreatment of target volumes (eg, prostate gland, seminal vesicles, or pelvic nodes). Intensity-modulated radiation therapy delivery requires expertise in treatment planning, set-up, alignment, and reproducibility, which are features that could be more readily available at high- vs low-volume centers. Second, clinical staff, including advanced practitioners and nurses, at high-volume centers may have more experience with managing acute toxic effects associated with aggressive local and systemic therapy, and adherence to treatment completion may subsequently be higher. Third, high-volume centers may more routinely use advanced imaging, such as magnetic resonance imaging or prostate-specific membrane antigen positron emission tomography, which may result in stage migration by ruling out metastatic disease and overall better outcomes compared with staging by conventional imaging alone. Furthermore, access to advanced molecular imaging during posttreatment surveillance may allow for earlier detection of disease recurrence during follow-up and improved salvage therapy.^35^ Positron emission tomography with computed tomography was not approved for initial prostate cancer staging until after the time period of this cohort study, yet its use in the posttreatment surveillance period was more widespread in the years immediately after 2016 and could impact salvage therapy and ultimately OS. Fourth, high-volume facilities are more likely to evaluate patients in a multidisciplinary setting, which leads to more aggressive, multimodal therapy for patients with higher risk disease.^36^ Treatment of patients with advanced prostate cancer requires close collaboration between urologists, radiation oncologists, medical oncologists, radiologists, and pathologists.^37^ Each member of this complex team contributes to timely diagnosis, optimal staging, early initiation of therapy, and posttreatment surveillance. It is plausible that high-volume centers more often have closer collaboration and workflows between these disciplines, including establishment of multidisciplinary clinics and tumor boards. Many of these characteristics are also representative of academic centers with specialized care; however, we observed the outcome of case volume and long-term survival even after adjusting for academic vs nonacademic facilities.

### Limitations

Our study has several limitations. First, given its retrospective design using observational data, selection bias and imbalances cannot be entirely mitigated through multivariable modeling and propensity score weighting. For example, men treated at low-volume centers were older, had greater comorbidity, lower socioeconomic status, and were less likely to be privately insured; all these factors may impact OS, which may not be fully accounted for by statistical measures. Second, the NCDB is a hospital-based registry that captures only patients treated at Commission on Cancer–accredited facilities; these results may not represent the entire cancer population of the US. However, given that 70% of all newly diagnosed cancer cases are reported to the NCDB each year, we believe this analysis reflects outcomes in facilities not captured by this registry. Third, outcome measures in the NCDB are limited to OS, so details regarding distant metastasis and cancer-specific survival are unavailable. However, we believe OS is the standard end point in men with VHR prostate cancer given the advanced nature of the disease with higher likelihood of distant metastatic progression and death compared with other localized prostate cancer. Fourth, toxicity and quality-of-life measurements are unavailable in the NCDB and could not be assessed; it is plausible that improved survival at high-volume centers is associated with more aggressive therapy and subsequent worse acute toxic effects and quality of life. Fifth, details regarding concomitant ADT, including drug type or duration, are not available in the NCDB and could not be accounted for despite being associated with long-term outcomes in this population.^38^ Along these lines, although NCDB data accuracy and quality are continually validated via data quality review, site surveys, and internal monitoring,^26^ a proportion of patient data may be internally inconsistent and could confound our results.^39^

## Conclusions

In this cohort study of patients with VHR prostate cancer treated with radiotherapy and ADT in the US, treatment at a facility with a high radiation case volume was associated with longer OS. Further studies should focus on identifying which factors unique to high-volume centers may be accounting for this benefit.

## Notes

Supplement 1. **eTable 1.** Overall Sample Distribution and Balance Check for Before and After IPSW **eTable 2.** Multivariable Cox Proportional Hazards Model for Overall Survival Stratified by Facility Type **eFigure.** Kaplan-Meier Overall Survival Plots for Patients Treated at High- vs Low-ACFV Centers, Stratified by Facility Type (Academic vs Nonacademic)

Supplement 2.
Data Sharing Statement
