# Supplementary material for: Radiation Facility Volume and Survival for Men With Very High-Risk Prostate Cancer Treated with Radiation and Androgen Deprivation Therapy
Source: JAMA Netw Open. 2023 Aug 8;6(8):e2327637. doi: 10.1001/jamanetworkopen.2023.27637 (PMC10410484; doi:10.1001/jamanetworkopen.2023.27637)
Supplement: Supplement 1. — eTable 1. Overall Sample Distribution and Balance Check for Before and After IPSW eTable 2. Multivariable Cox Proportional Hazards Model for Overall Survival Stratified by Facility Type eFigure. Kaplan-Meier Overall Survival Plots for Patients Treated at High- vs Low-ACFV Centers, Stratified by Facility Type (Academic vs Nonacademic) [file jamanetwopen-e2327637-s001.pdf]

Supplementary Online Content

Sebastian N, Goyal S, Liu Y, et al. Radiation facility volume and survival for men with very high-risk prostate cancer treated with radiation and androgen deprivation therapy. *JAMA Netw Open*. 2023;6(8):e2327637. doi:10.1001/jamanetworkopen.2023.27637

eTable 1. Overall Sample Distribution and Balance Check for Before and After IPSW

eTable 2. Multivariable Cox Proportional Hazards Model for Overall Survival Stratified by Facility Type

eFigure. Kaplan-Meier Overall Survival Plots for Patients Treated at High- vs Low-ACFV Centers, Stratified by Facility Type (Academic vs Nonacademic)

This supplementary material has been provided by the authors to give readers additional information about their work.

© 2023 Sebastian N et al. *JAMA Network Open.*

eTable 1. Overall Sample Distribution and Balance Check for Before and After IPSW

Covariate	Level	Study Sample Distribution Before	Study Sample Distribution After	Absolute Standardized Difference(ASD) Before	Absolute Standardized Difference(ASD) After
Average Cumulative Facility Volume (optimal cutoff)	Overall	24961 (100.0)	24940 (100.0)	-	-
	Low	18585 (74.5)	18603 (74.6)	-	-
	High	6376 (25.5)	6338 (25.4)	-	-
Age	< 65	12430 (49.8)	12424 (49.8)	0.032	0.002
	≥ 65	12531 (50.2)	12517 (50.2)	0.032	0.002
Race	White	19656 (78.7)	19575 (78.5)	0.051	0.017
	Black	4264 (17.1)	4334 (17.4)	0.007	0.018
	Other	1041 (4.2)	1031 (4.1)	0.088	0.001
Zip Code Median Income	>=$68,000	7823 (31.3)	7731 (31)	**0.252**	0.014
	$48,000-$67,999	6603 (26.5)	6580 (26.4)	0.092	0.007
	$38,000-$47,999	5884 (23.6)	5898 (23.6)	**0.122**	0.004
	<$38,000	4651 (18.6)	4732 (19)	0.072	0.02
Zip Code Percentage Without High School Degree	<7.0%	5999 (24)	6019 (24.1)	**0.124**	0.006
	7.0-12.9%	8310 (33.3)	8298 (33.3)	0.005	0.002
	13.0-20.9%	6515 (26.1)	6447 (25.9)	0.063	0.016
	>=21%	4137 (16.6)	4176 (16.7)	0.079	0.009
Insurance Type	Medicaid/Uninsured	2507 (10)	2561 (10.3)	**0.117**	0.02
	Private	6689 (26.8)	6657 (26.7)	**0.163**	0.007
	Medicare	15765 (63.2)	15723 (63)	0.081	0.006
Facility Type	Non-Academic/Research Program	17339 (69.5)	17267 (69.2)	**0.538**	0.009
	Academic/Research Program	7622 (30.5)	7673 (30.8)	**0.538**	0.009
Charlson-Deyo Comorbidity Score	0	20688 (82.9)	20712 (83)	**0.11**	0.01
	1	3294 (13.2)	3245 (13)	0.097	0.014
	2+	979 (3.9)	983 (3.9)	0.045	0.005

© 2023 Sebastian N et al. *JAMA Network Open.*

eTable 1. Overall Sample Distribution and Balance Check for Before and After IPSW

		Study Sample Distribution		Absolute Standardized Difference(ASD)	
**Covariate**	**Level**	**Before**	**After**	**Before**	**After**
AJCC Clinical T-stage	T1	9317 (37.3)	9276 (37.2)	0.073	0.005
	T2	9015 (36.1)	9002 (36.1)	0.028	0
	T3-4	6629 (26.6)	6663 (26.7)	**0.108**	0.006
PSA	<10	10143 (40.6)	10126 (40.6)	0.033	0.003
	10-20	5372 (21.5)	5311 (21.3)	0.003	0.012
	>20	9446 (37.8)	9504 (38.1)	0.036	0.014
Gleason score	6-7	1566 (6.3)	1586 (6.4)	0.071	0.003
	8-10	23395 (93.7)	23355 (93.6)	0.071	0.003
Total Radiation Dose (cat.)	<74	4936 (19.8)	5044 (20.2)	**0.19**	0.021
	>=74	20025 (80.2)	19896 (79.8)	**0.19**	0.021
Year of Diagnosis	>=2004, <=2009	7245 (29)	7392 (29.6)	**0.157**	0.025
	>2009, <=2011	5451 (21.8)	5437 (21.8)	0.034	0.001
	>2011, <=2014	8725 (35)	8622 (34.6)	0.074	0.013
	>2014, <=2016	3540 (14.2)	3489 (14)	**0.154**	0.016
Distance to Facility (miles)	>=0, <=4.1	6259 (25.1)	6212 (24.9)	**0.143**	0.013
	>4.1, <=9.1	6321 (25.3)	6407 (25.7)	0.021	0.02
	>9.1, <=19.8	6145 (24.6)	6214 (24.9)	0.031	0.017
	>19.8, <=2914.7	6236 (25)	6107 (24.5)	0.086	0.024

* The absolute standardized Difference (ASD) >= 0.1 is bold and indicates insufficient balance.

© 2023 Sebastian N et al. *JAMA Network Open.*

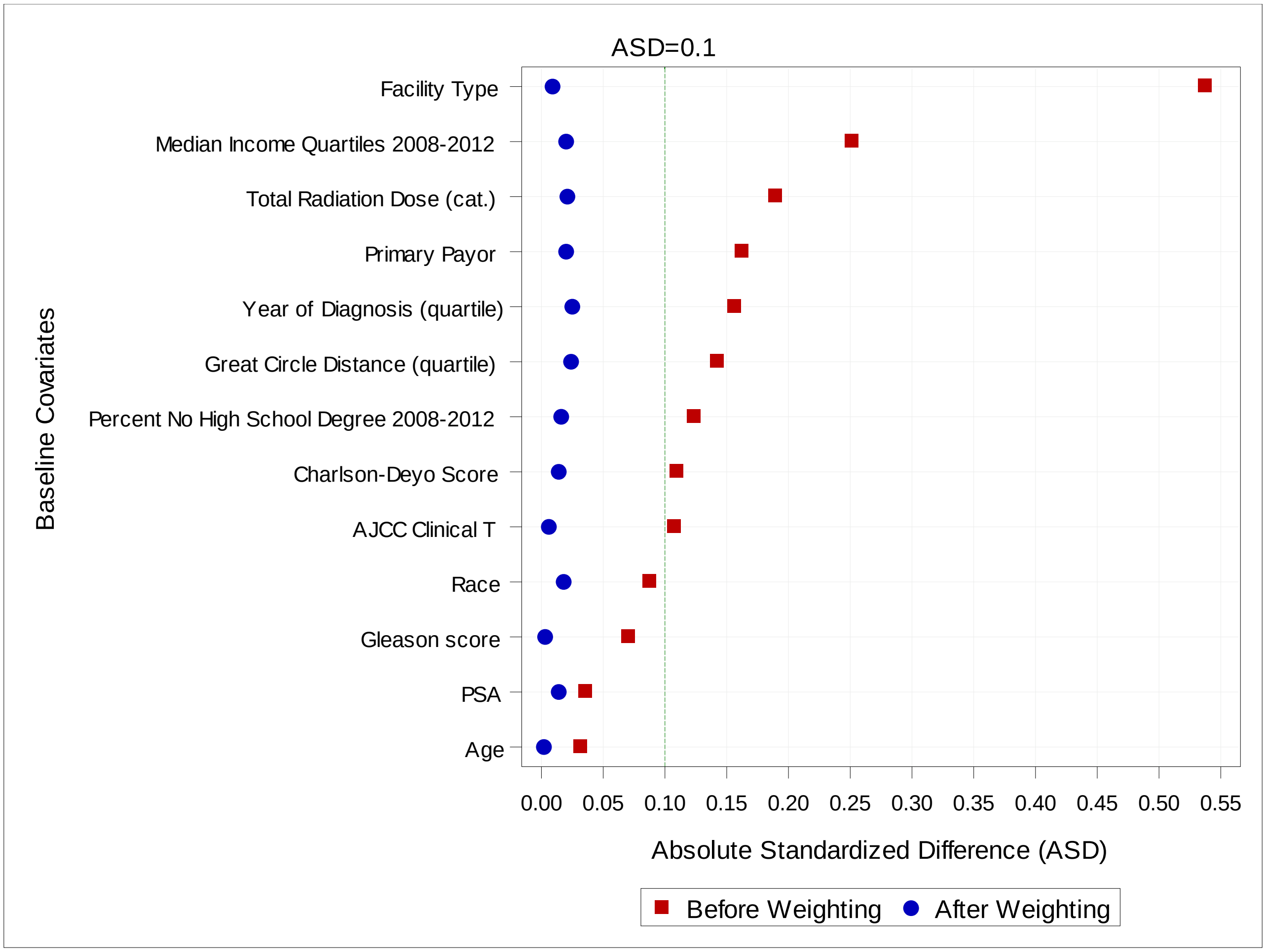

© 2023 Sebastian N et al. *JAMA Network Open.*

eTable 2. Multivariable Cox proportional hazards model for overall survival stratified by facility type

		OS		
**Covariate**	**Level**	**Hazard Ratio (95% CI)**	**HR P-value**	**Overall P-value**
**Comparisons Stratified by Facility Type:**	**Average Cumulative Facility Volume (optimal cutoff):**	-	-	0.39
Non-Academic/Research Program	High vs. Low	0.91 (0.84-0.98)	**0.01**	-
Academic/Research Program	High vs. Low	0.86 (0.78-0.95)	**0.003**	-

* Number of observations in the original data set = 25219. Number of observations used = 25126.
** Backward selection with an alpha level of removal of .05 was used. The following variables were removed from the model: Great Circle Distance (quartile), and Percent No High School Degree 2008-2012.
*** The estimated stratified treatment effect was controlled by: Age, Charlson-Deyo Score, Median Income Quartiles 2008-2012, Primary Payor, Race, Year of Diagnosis (quartile)

© 2023 Sebastian N et al. *JAMA Network Open.*

eFigure. Kaplan-Meier overall survival plots for patients treated at high- vs low-ACFV centers, stratified by facility type (academic vs nonacademic)

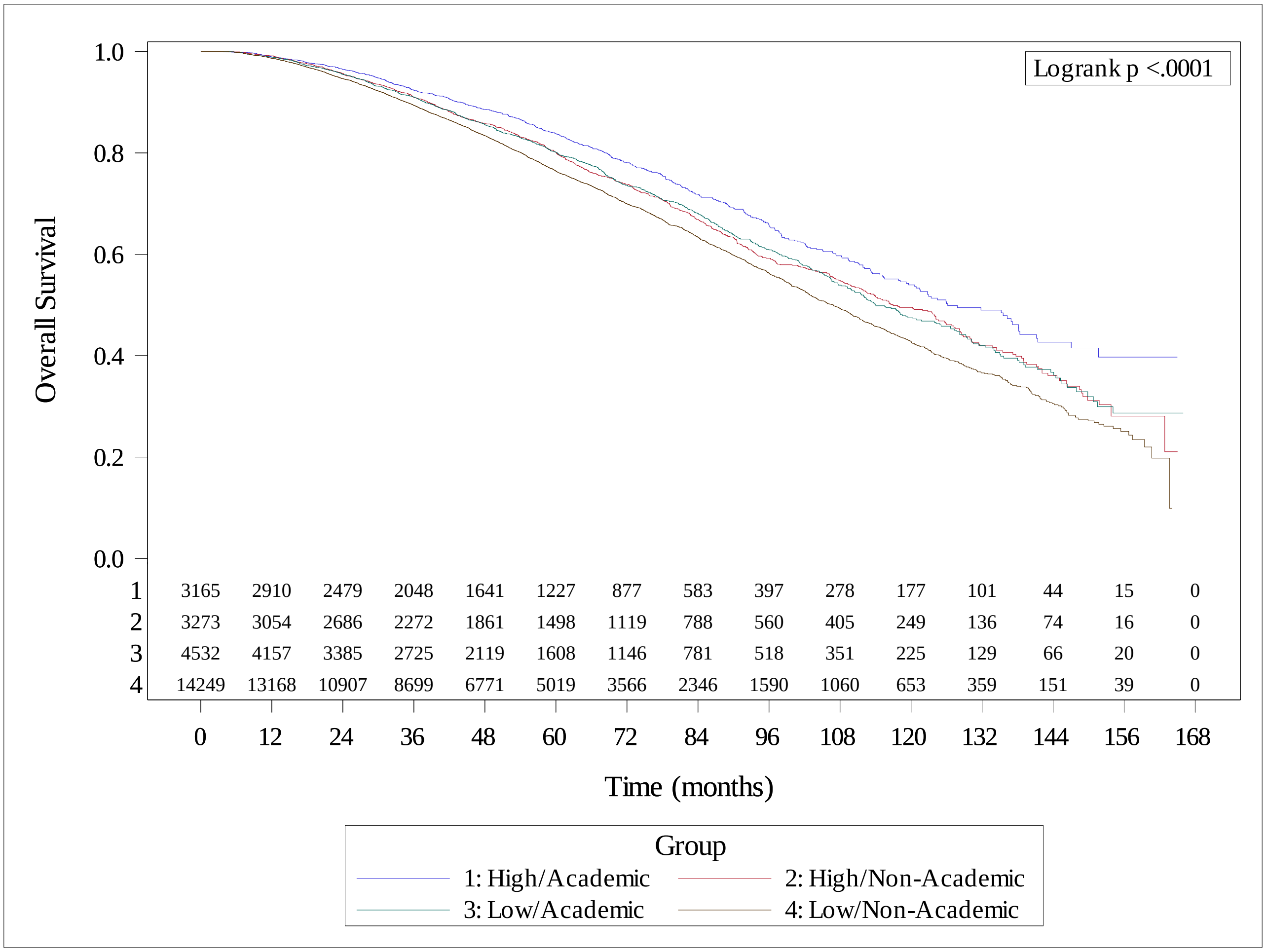

**Group**	**No. of Subject**	**Event**	**Censored**	**Median Survival (95% CI)**
High/Academic	3165	609 (19%)	2556 (81%)	126.2 (120.6, 138.2)
High/Non-Academic	3273	898 (27%)	2375 (73%)	117 (111.9, 124.7)
Low/Academic	4532	986 (22%)	3546 (78%)	114 (110, 121.8)
Low/Non-Academic	14249	3615 (25%)	10634 (75%)	106.8 (103.8, 109.4)

© 2023 Sebastian N et al. *JAMA Network Open.*
