# Supplementary material for: Radiation Facility Volume and Survival for Men With Very High-Risk Prostate Cancer Treated with Radiation and Androgen Deprivation Therapy
Source: JAMA Netw Open. 2023 Aug 8;6(8):e2327637. doi: 10.1001/jamanetworkopen.2023.27637 (PMC10410484; doi:10.1001/jamanetworkopen.2023.27637)
Supplement: Supplement 2. — Data Sharing Statement [file jamanetwopen-e2327637-s002.pdf]

Data Sharing Statement

Sebastian. Radiation Facility Volume and Survival for Men With Very High-Risk Prostate Cancer Treated with Radiation and Androgen Deprivation Therapy. *JAMA Netw Open*. Published August 08, 2023. doi:10.1001/jamanetworkopen.2023.27637

Data
Data available: No

Additional Information
Explanation for why data not available: The National Cancer Database is available to those institutions who participate in the Commission-on-Cancer consortium.
